# Intravascular clusters of T cells following chimeric antigen receptor T cell therapy

**DOI:** 10.1002/jha2.395

**Published:** 2022-02-04

**Authors:** Naokazu Nakamura, Momoko Nishikori, Marina Tsujimura, Yuki Kageyama, Daisuke Akiyama, Akihiko Yoshizawa, Akifumi Takaori‐Kondo

**Affiliations:** ^1^ Department of Hematology/Oncology Graduate School of Medicine Kyoto University Kyoto Japan; ^2^ Department of Diagnostic Pathology Kyoto University Hospital Kyoto Japan; ^3^ Department of Hematology Yokkaichi Municipal Hospital Yokkaichi Japan

A 52‐year‐old male was diagnosed with diffuse large B‐cell lymphoma (DLBCL) with BCL2, BCL6, and MYC translocations (triple hit lymphoma). In addition to nodal, splenic, and gingival lesions, bone marrow was extensively involved with lymphoma cells (up to 90% of the nucleated cells). Although he received eight cycles of dose‐adjusted (DA)‐EPOCH‐R (etoposide, prednisolone, vincristine, cyclophosphamide, doxorubicin, and rituximab), his lymphoma recurred shortly after the completion of planned therapy. His disease was not controlled by additional three lines of chemotherapy, but as there seemed no other effective treatment options, he proceeded to tisagenlecleucel therapy at his own wish.

On the day of chimeric antigen receptor (CAR)‐T cell infusion, lymphoma cells still remained at 56% of total peripheral leukocytes by smear examination, whereas normal leukocytes and platelets were severely suppressed by preceding chemotherapy (total leukocyte count 70 /μl, platelet count 16 × 10^3^ /μl). High fever began to appear shortly after the infusion, and nasal cannula oxygen therapy was started on day 2. In response to a diagnosis of cytokine release syndrome (CRS), four doses of tocilizumab were administered from day 2 to 3, but the symptoms did not improve. He developed immune effector cell‐associated neurotoxicity syndrome grade 2 and hypotension requiring vasopressor on day 4, and dexamethasone was initiated. In spite of intensive care, he died of rapidly progressive metabolic acidosis on day 5.

Autopsy demonstrated fluid retention and visceral congestion, which were considered to be caused by severe CRS. CD19‐negative and CD20‐positive B cells were present in the bone marrow and considered as residual lymphoma cells that lost CD19 expression after CAR‐T cell therapy. Notably, a 2.0 × 1.0 mm‐sized cellular cluster was found in the pulmonary artery (Figure 1), and a smaller cluster in the pulmonary vein. These clusters were not obstructive and the vessel lumens were preserved. The clusters were mostly consisted of CD4‐ and CD8‐positive T cells, while the content of CAR‐T cells could not be evaluated. A few, deformed CD20‐positive B cells were detected in the cluster, but they lacked CD19 expression as B cells in the bone marrow.

**FIGURE 1:**
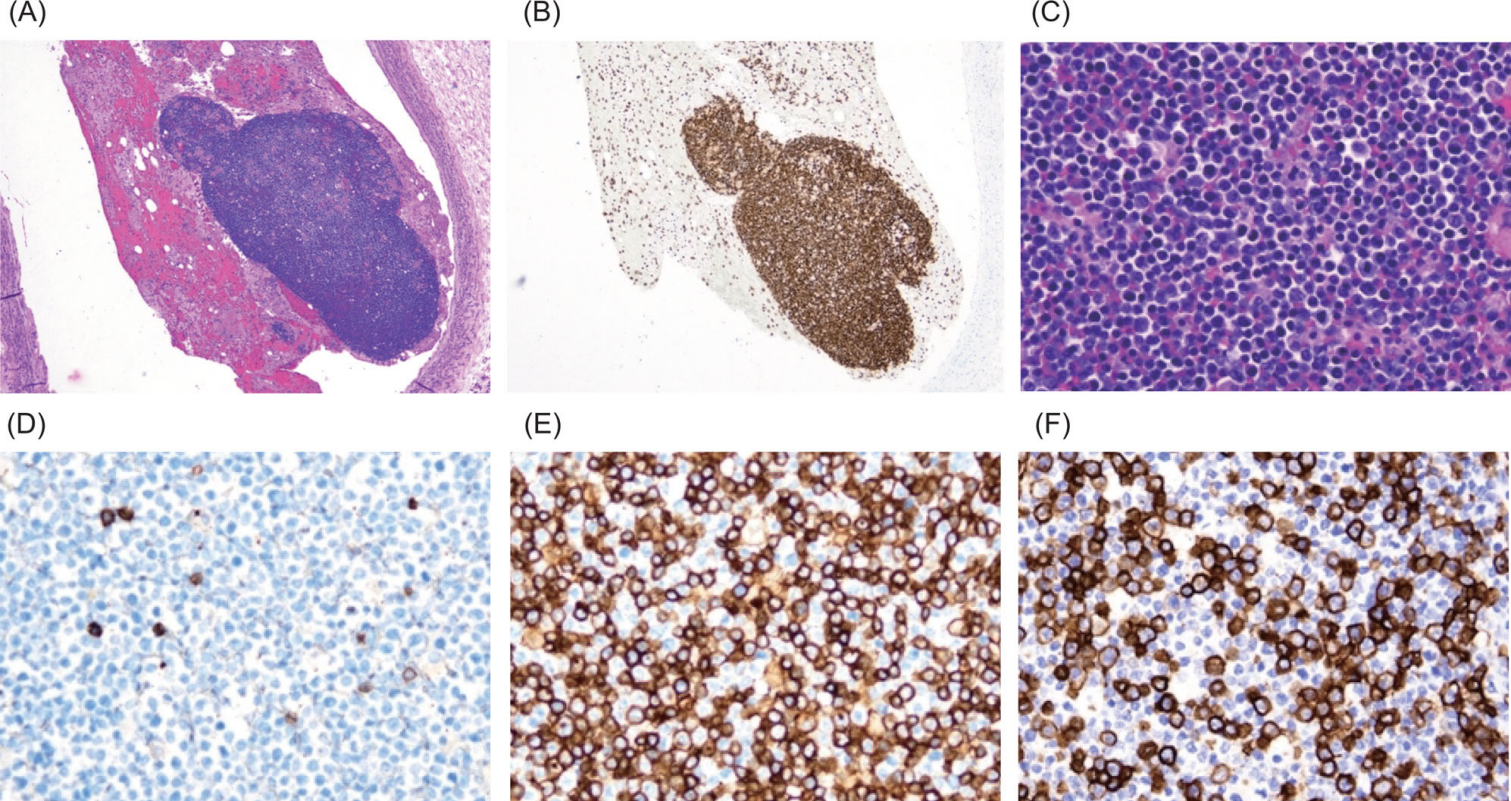
A T cell cluster found in the pulmonary artery of the patient. (A) Hematoxylin and eosin (H&E) and (B) CD3 (40x); (C) H&E, (D) CD20, (E) CD4, and (F) CD8 (200x)

CRS is a frequent adverse effect of CAR‐T cell therapy characterized by the elevation of various inflammatory cytokines, such as interleukin (IL)‐6, IL‐1, and interferon‐gamma [1, 2]. CRS often leads to coagulopathy by triggering endothelial damage, platelet aggregation, and activation of the coagulation cascade [3, 4, 5]. The patient was at high risk for CRS due to high tumor burden, thrombocytopenia, and elevated ferritin level (12,293 ng/ml on day 3) [1], and rapid expansion of CAR‐T cells probably induced severe cytokine storm and enhanced coagulation activation. What is notable is the formation of intravascular clusters made up of T cells, which has not been previously demonstrated clinically. In a mouse model of CAR‐T cell administration to aggressive B‐cell lymphoma carrying Eμ‐MYC transgene, Cazaux et al. reported that large cellular clusters consisting of CAR‐T cells and malignant B cells were generated in the circulation, and consequently trapped in the lungs [6]. We assume that circulating lymphoma cells strongly induced T cell proliferation and aggregation in vessels in our patient, in a similar mechanism to this mouse lymphoma model. Although it was unlikely that these clusters directly caused his death, we would like to highlight this rare but possible finding in patients treated with CAR‐T cell therapy.

## CONFLICT OF INTEREST

The authors declare no conflict of interest.
